# Resting-state functional connectivity and amyloid burden influence longitudinal cortical thinning in the default mode network in preclinical Alzheimer’s disease

**DOI:** 10.1016/j.nicl.2020.102407

**Published:** 2020-09-02

**Authors:** Olivia L. Hampton, Rachel F. Buckley, Lyssa K. Manning, Matthew R. Scott, Michael J. Properzi, Cleofé Peña-Gómez, Heidi I.L. Jacobs, Jasmeer P. Chhatwal, Keith A. Johnson, Reisa A. Sperling, Aaron P. Schultz

**Affiliations:** a Department of Neurology, Massachusetts General Hospital, Boston, MA 02114, USA; b Melbourne School of Psychological Science, University of Melbourne, VIC 3010, Australia; c Department of Radiology, Massachusetts General Hospital/Harvard Medical School, Boston 02114, MA, USA; d Faculty of Health, Medicine and Life Sciences, School for Mental Health and Neuroscience, Alzheimer Centre Limburg, Maastricht University, Maastricht 6200, The Netherlands; e Center for Alzheimer Research and Treatment, Department of Neurology, Brigham and Women's Hospital, Boston, MA 02115, USA

**Keywords:** Functional connectivity, Amyloid, Default mode network, Alzheimer’s disease

## Abstract

Proteinopathies are key elements in the pathogenesis of age-related neurodegenerative diseases, particularly Alzheimer’s disease (AD), with the nature and location of the proteinopathy characterizing much of the disease phenotype. Susceptibility of brain regions to pathology may partly be determined by intrinsic network structure and connectivity. It remains unknown, however, how these networks inform the disease cascade in the context of AD biomarkers, such as beta-amyloid (Aβ), in clinically-normal older adults. The default-mode network (DMN), a prominent intrinsic network, is heavily implicated in AD due to its spatial overlap with AD atrophy patterns and tau deposition. We investigated the influence of baseline Aβ positron emission tomography (PET) signal and intrinsic DMN connectivity on DMN-specific cortical thinning in 120 clinically-normal older adults from the Harvard Aging Brain Study (73 ± 6 years, 58% Female, CDR = 0). Participants underwent ^11^C Pittsburgh Compound-B (PiB) PET, ^18^F flortaucipir (FTP) PET, and resting-state MRI scans at baseline and longitudinal MRI (3.6 ± 0.96 scans; 5.04 ± 0.8 years). Linear mixed models tested relationships between baseline PiB and DMN connectivity on cortical thinning in a composite of DMN regions. Lower DMN connectivity was associated with faster cortical thinning, but only in those with elevated baseline PiB-PET signal. This relationship was network specific, in that the frontoparietal control network did not account for the observed association. Additionally, the relationship was independent of inferior temporal lobe FTP-PET signal. Our findings provide evidence that compromised DMN connectivity, in the context of preclinical AD, foreshadows neurodegeneration in DMN regions.

## Highlights

• Low baseline default-mode integrity and high amyloid presage future network atrophy.
• The high amyloid group drives the within-network atrophy effect.
• Alzheimer’s disease-related biomarkers didn’t explain the high amyloid group’s effect.
• Results support links between functional and cortical integrity and connectivity as a protective factor.

## 1 Introduction

Although β-amyloid (Aβ) is a hallmark pathologic trait of Alzheimer’s disease (AD), simply knowing levels of Aβ does not sufficiently predict development of dementia. Additional biomarkers including tau pathology (Brier et al., 2016, Gordon et al., 2018, Jack et al., 2019, Mattsson et al., 2019, Sperling et al., 2019), neurodegeneration (Mormino et al., 2014a, Jack et al., 2019, Mattsson et al., 2019), cardiovascular risk (Vemuri et al., 2015, Rabin et al., 2018), and functional connectivity (Buckley et al., 2017, Van Hooren et al., 2018) can help better predict risk for future decline in the context of elevated Aβ burden. Regarding connectivity, a growing body of evidence has implicated connectivity within the default-mode network (DMN) as particularly vulnerable to AD pathology (Buckner et al., 2005, Sperling et al., 2009, Jones et al., 2016, Palmqvist et al., 2017, Chhatwal et al., 2018). DMN cortical regions overlap with late stage atrophy signatures of AD (Dickerson et al., 2009), sites of high amyloid burden (Mormino et al., 2011, Buckner et al., 2009), and a posterior subset of DMN regions co-locate with patterns of neurofibrillary tau accumulation (Johnson et al., 2016, Franzmeier et al., 2019, Ossenkoppele et al., 2019, Li et al., 2019). Recent evidence suggests lower functional connectivity negatively impacts cognitive outcomes over time in individuals with elevated Aβ burden (Buckley et al., 2017). The longitudinal neurodegenerative implications of impaired DMN connectivity and elevated Aβ in clinically-normal older adults, however, are still unelucidated. If network integrity relates to neurodegeneration and future cognition, then we predict network coherence should be indicative of future atrophy, particularly in the context of amyloid, which is associated with neurodegenerative processes.

To investigate this question, we examined the relationship of baseline DMN connectivity and amyloid with longitudinal default mode neurodegeneration in healthy older adults. Specifically, we tested the hypothesis that lower DMN connectivity and higher Aβ burden at baseline would interact to influence faster cortical thinning in a DMN cortical composite. We also investigated the extent to which cortical thinning in the DMN was specifically associated with DMN connectivity by controlling for thinning and connectivity measures from the frontoparietal control network and tau-PET, as higher levels of tau accumulation spatially intersect with thinner cortex in network hubs of the DMN (LaPoint et al., 2017, Mak et al., 2018, Ossenkoppele et al., 2019, Li et al., 2019).

## 2 Materials and methods

### 2.1 Participants

We included 120 clinically normal participants, aged 62.75–88.25 years at baseline (SD = 6.0), from the Harvard Aging Brain Study (HABS) who had at least two MRI scans and a baseline PiB-PET scan (see Table 1). Participants received MRI scans once every 2–3 years (Dagley et al., 2017). Structural MRI spanned an average of 5.04 years (range 3.69–6.96 years) over an average of 3.6 visits (range 2–8 visits). Four participants had 2 scans, 56 participants had 3, and 60 had 4 or more scans. PiB sessions occurred on average 0.25 year (SD = 0.42, range 0–2.78 years) from the individual’s first MRI session. As part of HABS, all participants at baseline had to score 0 on the Clinical Dementia Rating scale, greater than 25 on the Mini-Mental Examination, and<11 on the Geriatric Depression Scale. Most recent diagnoses based on follow-up neuropsychological visits concluded 4 subjects were diagnosed with dementia, and 10 were diagnosed with MCI.

**Table 1:** Demographic information.

N	Overall 120		low PiB 87		high PiB 33		*t*-test, Chi-square test	
	Mean	SD	Mean	SD	Mean	SD	Effect size	*p*-value
Age	73.35	6.0	72.93	6.3	74.5	4.8	1.29	0.20
Education (yrs)	16.02	2.9	16.1	3.0	16.6	2.7	0.84	0.40
PiB FLR DVR	1.18	2.9	1.08	0.1	1.46	0.1	18.6	< 0.0001
Sex # F (% F)	70 (58)		52 (59.8)		18 (54.5)		0.03	0.67
APOE e4 # (% +)	37 (30.8)		17 (19.5)		21 (63.6)			
AMNART VIQ	122.2	7.9	121.9	8.0	122.9	8.0	0.61	0.54
# MRI visits	3.6	0.96	3.6	0.95	3.6	1.0		
Time span (yrs)	5.04	0.8	5.1	0.8	4.9	0.6	1.30	0.20

Note: PiB FLR DVR = Pittsburgh Compound-B distribution volume ratio of frontal, lateral, and retrosplenial tracer uptake; PiB positivity determined by FLR cutoff of 1.186 derived from a Gaussian mixed model; APOE = Apolipoprotein E; AMNART VIQ = American National Adult Reading Test Verbal IQ.

### 2.2 Experimental Design: MRI

MRI scans were collected on one of two matched 3 T Trio Tim scanners with a 12-channel head coil at the Martinos Center for Imaging in Charlestown, Massachusetts. Scanner noise was attenuated using foam earplugs, and foam padding was placed around the head to limit head movement. T1-weighted magnetization-prepared rapid gradient echo (MPRAGE) scans had the following parameters: TR/TE/inversion time 2,300/2.95/900 ms, flip angle 9°, 1.1 × 1.1 × 1.2 mm resolution. The T1 scans were processed with FreeSurfer v6.0 (FS6). After initial FS6 recon, each scan was manually quality-checked for movement artifact and segmentation accuracy. Errors to the pial surface and white matter were adjusted by removing non-brain matter voxels, placing control points, and reprocessing cases until accurate segmentation results were obtained or the scan was deemed unusable. All scans were run through the FS6 longitudinal pipeline. The longitudinal pipeline creates temporally-unbiased templates for each person based on an average of all timepoints from the corresponding participant (Reuter et al., 2012). Each scan is resampled to the median space to reduce random variability between timepoints and to improve sensitivity. Each template was quality checked according to previously published methods to ensure accuracy of pial and white matter surfaces (Dagley et al., 2017). In accordance with the FS longitudinal pipeline (Reuter et al., 2012), any poor template was monitored by 1) tracing to the contributing cross-sectional scans and performing additional quality edits before recreating the template, 2) editing the template directly until satisfaction, or 3) deeming the subject’s data unusable. FcMRI data were acquired using a gradient-echo echoplanar imaging sequence sensitive to BOLD contrast. Whole-brain coverage, including the cerebellum, was acquired while aligned parallel to the anterior/posterior commissure using the following parameters: TR/TE 3000/30 ms; flip angle, 85°; field of view, 216x216 mm; matrix, 72x72; and 3x3x3 mm voxels; 124 volumes were acquired in one or 2 6-minute runs (including 4 dummy volumes; 12 s which were subsequently discarded). Instructions were to lie still, remain awake, and keep eyes open and focus on a fixation cross projected on a screen at the head of the bore.

Each 6-minute baseline resting state fMRI scan was realigned, resliced, and then morphometrically aligned to the T1 image using SPM12 (Function Imaging Laboratory, Wellcome Department of Cognitive Neurology, London, UK), spatially normalized to MNI space using the warping field derived on the T1 image, smoothed with a 6 mm Gaussian smoothing kernel, and temporally filtered with a fourth order Butterworth bandpass filter (0.08 Hz-0.1 Hz).

Tissue probability maps from the T1 (derived with the SPM12 unified segmentation/normalization routine) were used to identify voxels with a maximum tissue probability belonging to white matter, cerebral spinal fluid, or bone. For each of these three tissue classes we performed a temporal PCA and retained the top 5 components from each tissue class. We also used the 6 movement parameters from the realignment step plus first derivatives and squared terms yielding a total of 33 nuisance regressors. All nuisance regressors were temporally filtered in the same manner as the resting state scan. We then performed a PCA on the 33 z-scored regressors and retained the number of PCs needed to account for 90% of the variance in the nuisance regressor set within each individual. This PCA step was performed to reduce the dimensionality of the nuisance regressor set and avoid overfitting of the nuisance regressors to the resting state fMRI data. For our sample, an average of 7.7 ± 2.4 nuisance regressors were then removed from each individual fMRI session.

**Fig. 1:**
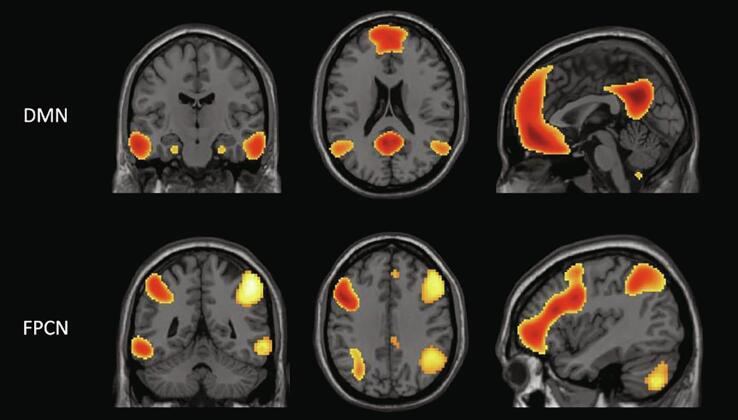
Network template maps – Network template maps used for connectivity measures and cortical composites based on TBR methods (adapted from Schultz et al., 2014). DMN = Default Mode Network; FPCN = Frontoparietal Control Network.

**Fig. 2:**
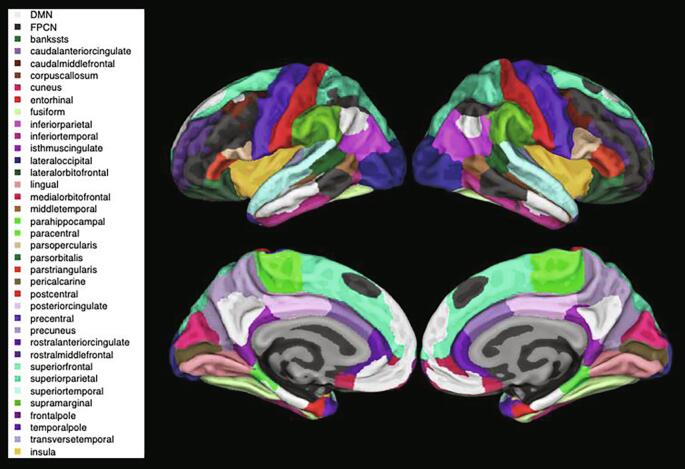
Network composite volumetric maps – Overlap of the TBR-derived DMN (in white) and FPCN (in black) network composites over the Desikan-Killany atlas (Desikan et al., 2006). The full list of atlas brain regions is listed on the left. DMN = Default Mode Network; FPCN = Frontoparietal Control Network.

We derived fcMRI maps for the default mode network (DMN) and frontoparietal control network (FPCN) using our previously published Template Based Rotation (TBR) method (Schultz et al. 2014; Fig. 1). The FPCN measure was an average of measures from the Left and Right FPCN templates. For an exploratory analysis, we extracted the signal from the overlapping FPCN voxels within the DMN connectivity maps to create an FPCN-within-DMN measure as well as a DMN-within-FPCN measure to explore inter-network connectivity (see Fig. 2).

TBR maps variance from a given subject's fMRI run to a set of *a priori* template maps. The process produces a least squares fit to the template maps via a weighted linear summation of functional volumes. The weights associated with each functional volume can be interpreted as the time series that best recapitulates the spatial pattern in the templates. As with spatial group independent component analysis (ICA), when using TBR there is no explicit requirement to clean data in advance of analysis. ICA can be written in matrix algebra form as IC = X•M where IC is the independent components (m-voxels by n-components), M is the unmixing matrix (m-volumes by n-components), and X is the empirical data (m-voxels by n-volumes). Starting with only X, M must be discovered which then provides IC. If IC is already known, then the derivation of M is a straightforward algebraic problem that can be solved as M = X^−1^ •IC, which is precisely the TBR formulation (where X^−1^ is the pseudo-inverse of the matrix X). The only assumption is that a given IC, derived on an independent dataset, is a sufficiently close approximation to a true source in X, and is sufficiently independent of nuisance variance sources. This provides TBR with an implicit ability to perform blind source/signal separation even when not explicitly modeling all source’s variance. Complete details can be found in Schultz et al., 2014.

Template maps were derived from a large independent sample of 675 young subjects from the Genome Superstruct project which utilized identical functional sequences. The derivation utilized factor rotation (orthomax rotation) on group level principal components to produce 20 spatial maps. These templates have been used in multiple prior publications. Complete details can be found in Schultz et al., 2014, and both template maps and relevant code for TBR are publicly available at mrtools.mgh.harvard.edu.

TBR connectivity measures for each participant and network were computed using the average correlation strength of all brain voxels within the network mask (greater than 40% maximum value in the corresponding template map), resulting in a whole network connectivity measure. Following functional connectivity analysis and extraction of network measures for all subjects we regressed out all between subject variance associated with movement (mean frame-to-frame displacement), and data dimensionality (number of principal components) utilized by the TBR algorithm (see Schultz, 2014 for details). As TBR can perform implicit data scrubbing by down-weighting specific volumes, no scrubbing is required (Schultz et al., 2019).

We made a group level template space version of the PETsurfer GTM segmentations (Greve et al., 2013), using the spatial normalization provided by the SPM12 unified segmentation/normalization algorithm on the T1 MPRAGE images. The group level atlas coded each voxel as the most frequently assigned label across 270 baseline MPRAGE scans from HABS. We then took the functionally defined regions of interest from Shaw et al., 2015, which were based on the 20 component parcellation from Schultz et al., 2014 (the same templates used in the present report), resampled each node mask to 0.5 mm resolution using cubic interpolation. Any voxel in the resampled space that contained a resampled mask value greater than 0.25 and that lay within the group level gray matter cortical partition was assigned the corresponding label. Cortical voxels that were not part of a functional node were assigned to an undefined label.

We then took this high resolution map of functional parcels specific to the group level MNI space gray matter partition and mapped it to fsaverage using a custom algorithm whereby for each fsaverage vertex we found all voxels that were within 2 mm of the vertex, and then assigned the vertex the value of the most frequently occuring label. These surface maps were then used to create an annotation file for the fsaverage subject, which were then mapped onto each subject’s native surface in order to obtain measures of cortical thickness associated with default mode and fronto-parietal control networks for each subject at each timepoint.

### 2.3 Experimental Design: PET

Full acquisition method details regarding ^11^C PiB-PET data collection in HABS are outlined in Johnson et al., 2013. In summary, PiB-PET images were acquired with an 8.5–15 mCI bolus injection with a 1-hour dynamic acquisition over 69 volumes (12 × 15 s, 57 × 60 s). PET images were subjected to reconstruction and attenuation correction, evaluated for head motion, and co-registered to the participant’s T1-weighted image using 6 degrees of freedom rigid body registration. For each participant, we calculated summary distribution volume ratio (DVR) of frontal, lateral, and retrosplenial (FLR) tracer uptake, using the average uptake across FreeSurfer defined ROI Desikan-Killany atlas (Desikan-Killany et al., 2006): precuneus, rostral anterior cingulate, medial orbito-frontal, superior frontal, rostral middle frontal, inferior parietal, inferior temporal, and middle temporal regions from both hemispheres. These target regions were then referenced to cerebellar grey matter signal. For our additional analyses, we dichotomised our sample according to a previously published PiB thresholding method (Mormino et al., 2014b; high PiB [n = 33] and low PiB [n = 87]).

For tau-PET measures, data were acquired using a Siemens CTI ECAT HR + scanner (3-dimensional mode, 63 image planes, 15.2 cm axial field of view, 5.6 mm trans axial resolution, and 2.4 mm slice interval). The scan took place in 4 × 5 min frames for 80–100 min after a 9.0–11.oCi bolus injection of ^18^F Flortaucipir PET (FTP-PET), prepared with a radiochemical yield of 14 ± 3% and specific activity of 216 ± 60 GBq/μmol at the end of synthesis as previously reported (Johnson et al., 2016), and validated for human use (Shoup et al., 2013). All PET images were reconstructed and attenuation corrected, and each frame was evaluated to verify adequate count statistics and absence of excessive head motion.

To evaluate the anatomy of cortical FTP-PET binding, PET scans were rigidly coregistered to the individual’s T1-weighted MRI scan closest in time to the FTP-PET scan using SPM12. FTP-PET data were partial-volume corrected using the Geometric Transfer Matrix (GTM) method (Rousset et al., 1998) using the FS6 PET-Surfer implementation (Greve et al., 2013). FTP signal was measured in the bilateral inferior temporal gyrus as a standardized uptake value ratio (SUVR), using FS6 defined cerebellar gray as the reference region. We used inferior temporal tau signal as a proxy for AD-specific neocortical tau spread, which has been associated with cognitive decline (Hanseeuw et al., 2017, Sperling et al., 2019). Due to the relatively recent availability of FTP-PET, FTP-PET scans were not collected at baseline, but approximately in the middle of the MRI timeline (average 3.22 years from first MRI, SD: 1.36 years).

### 2.4 Statistical analyses

For all analyses, we used the *fitlme* statistical package in MATLAB R2017. We ran a series of linear mixed effect models with intercept and slope included as random factors to examine the influence of baseline DMN connectivity and continuous PiB on cortical thinning. Network connectivity values were controlled for nuisance regressors for each individual and demeaned based on the whole dataset before analyses. In all models, we used time-varying DMN cortical thickness as the outcome variable and included age, sex, and years of education as covariates. For our main analysis, we used the following model:

DMN composite thickness ~ DMN FC* PiB*time + Covariates*time + (time|subject)

To further investigate our findings, we ran the same analysis in 2 groups stratified by high and low PiB DVR without the PiB term in the model in order to observe the effect of baseline DMN on atrophy within each PiB group. We ran additional models to test specific *post hoc* hypotheses exclusively within the high PiB group to examine the influence of FPCN connectivity, time-varying FPCN network composite thickness, or inferior temporal tau-PET measurements on DMN composite cortical thinning in order to establish the specificity of DMN connectivity on DMN atrophy. PiB, FTP, and functional connectivity (FC) values were treated as continuous variables and demeaned before analyses. As the majority of statistical tests were post-hoc tests to unpack the primary DMN FC:PiB:time interaction we did not correct for multiple comparisons.

## 3 Results

### 3.1 DMN node thinning over time is associated with baseline DMN connectivity and higher PiB

**Fig. 3:**
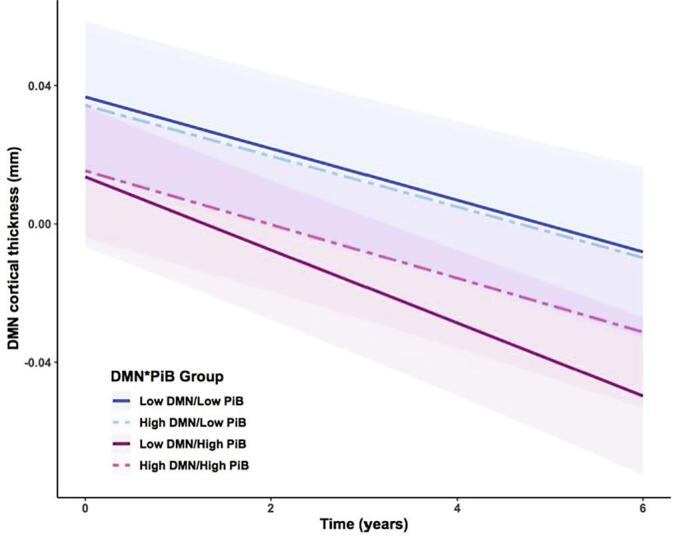
DMN composite thinning by baseline DMN and PiB: Predicted values from LME model analyzing the effect of baseline DMN and PiB signal on thinning in the DMN over time. Note: DMN and PiB signal values were median split in this diagram. We observed faster rates of thinning in the DMN composite in individuals with lower baseline DMN connectivity, but primarily in individuals who also have higher baseline PiB signal (solid red line). (For interpretation of the references to colour in this figure legend, the reader is referred to the web version of this article.)

**Fig. 4:**
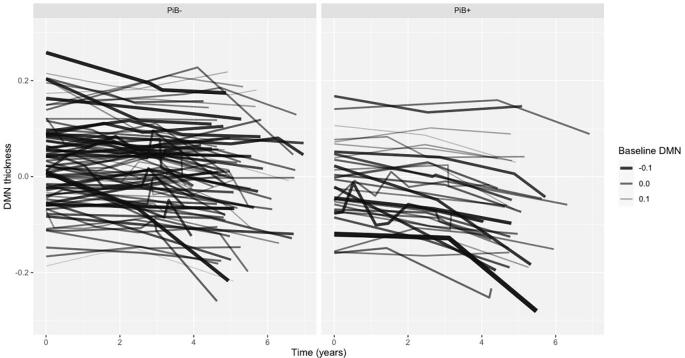
Spaghetti plots of raw data by PiB group: Each line depicts a single participant and the demeaned DMN composite thickness over time. Line aesthetics have continuous thickness and transparency values based on their continuous baseline DMN connectivity measure. Darker, thicker lines indicate lower baseline DMN values, while thinner, lighter lines indicate higher baseline DMN values. The panels are split by PiB- group (N = 87) and PiB + group (N = 33).

We observed a significant interaction between baseline DMN connectivity and PiB on cortical thinning in the DMN composite (*t*(421) = 2.43, *p* = 0.015, *d* = 0.24; see Table 2 for full statistical results). Specifically, lower DMN connectivity and higher PiB levels at baseline were associated with increased thinning in the DMN cortical composite over time (see Fig. 3). Raw DMN composite thickness data over time per participant is depicted in Fig. 4. To confirm our findings using an alternate atlas parcellation, we ran our main LME model interacting DMN functional connectivity and PiB signal from baseline to predict longitudinal thinning in the DMN composite based on Yeo et al., 2011. Similar with the TBR-derived DMN composite, we observed a significant negative interaction between baseline DMN connectivity and PiB signal (p = 0.024, t(421) = 2.27, d = 0.22). This result is comparable to, but slightly weaker than, the outcome using the TBR-derived DMN thickness composite.

**Table 2:** Results of DMN composite thinning by DMN connectivity and PiB in all participants and in each PiB group.

	DMN CT	DMN CT (PiB + )	DMN CT (PiB-)
PiB*Time	*t*(421) = −1.94; *p* = 0.053		
DMN*Time	*t*(421) = 1.69; *p* = 0.09	***t*(108) = 4.08; *p* = 0.00009**	*t*(307) = 0.24; *p* = 0.81
PiB*DMN	*t*(421) = 0.41; *p* = 0.68		
PiB*DMN*Time	***t*(421) = 2.43; *p* = 0.015**		

Note: DMN = Default Mode Network; CT = cortical thickness; PiB = Pittsburgh Compound-B; Age, sex, and years of education included as covariates.

Next, we stratified the model into high and low PiB groups based on the methods outlined above and removed the PiB term from the model. We found the effect of lower DMN connectivity at baseline in the high PiB group was significantly associated with increased thinning in the DMN composite (*t*(108) = 4.08, *p* = 0.00009, *d* = 0.78), but not in the low PiB group (*t*(307) = 0.24, *p* = 0.81, *d* = 0.03). Considering the high PiB group drove the effect of DMN connectivity on DMN thinning, we conducted additional analyses only in the high PiB group to further explore the relationship.

### 3.2 In high PiB individuals, the influence of baseline DMN connectivity on DMN composite thinning is not fully explained by other AD-related biomarkers

In additional analyses, we included other variables to the high PiB DMN model known to be associated with AD-related decline. First, we considered frontoparietal control network (FPCN) connectivity (Buckley et al., 2017) in addition to the DMN connectivity variable in order to investigate the specificity of within-network connectivity to atrophy. In the high PiB group, we found that the FPCN connectivity was not independently associated with cortical thinning in the DMN (*t*(1 0 6) = 0.24, *p* = 0.81, *d* = 0.05), while the influence of DMN connectivity became attenuated, but remained significant (*t*(1 0 6) = 2.48, *p* = 0.01, *d* = 0.48). To explore the relationship of inter-network connectivity and PiB on thinning in the DMN, we used an FPCN-within-DMN measure and a DMN-within-FPCN measure in additional models in the full sample but found no significant effect.

Next, we included time varying cortical thickness in the FCPN as an independent variable in addition to DMN connectivity to determine whether the observed atrophy in the DMN could be explained by changes in cortical thickness in regions associated with other cognitive networks. The DMN by time interaction remained significantly associated with DMN cortical thinning (*t*(1 0 7) = 2.36, *p* = 0.02, *d* = 0.46), even though the FPCN composite thickness variable was also strongly related to DMN thinning (*t*(1 0 7) = 12.2, *p* < 0.00001, *d* = 2.35).

Last, we investigated if inferior temporal (IT) tau-PET signal influenced the association between baseline DMN connectivity and cortical thinning in the DMN. We included IT FTP-PET by time as a covariate in the high PiB DMN model; even with the significant tau by time term in the model (*t*(106) = -3.73, *p* = 0.0003, *d* = -0.73), the effect of lower DMN connectivity on increased DMN atrophy in the high PiB group remained significant (*t*(106) = 3.67, *p* = 0.0004, *d* = 0.71). We informally investigated the three-way interaction of IT tau, DMN connectivity, and time on DMN thinning in the high PiB group but observed no significant effect. We dismissed these results, as we are limited by our sample size and the temporal incongruence in data collection of the tau-PET and fcMRI. When all three of these variables (FPCN connectivity, FPCN thickness, and IT tau) are included in the high PiB DMN model, the DMN by time interaction was no longer statistically significant, but still trended in the same direction (*t*(103) = 1.36, *p* = 0.18, *d* = 0.27).

## 4 Discussion

### 4.1 Discussion of results

We found that in the presence of elevated baseline Aβ burden, lower baseline DMN connectivity was associated with faster longitudinal atrophy within DMN associated regions. We observed that the effect was restricted to high Aβ individuals, and that the effect was independent of inferior temporal FTP-PET signal, baseline FPCN connectivity, or longitudinal change in FPCN thickness. Further, we did not observe an association between PiB-PET signal and DMN fcMRI strength, suggesting that lower DMN fcMRI at baseline is not a simple consequence of amyloid pathology. This suggests that functional connectivity may represent a proxy of resilience to the AD pathological cascade (Arenaza-Urquiljo and Vemuri, 2018, Franzmeier et al., 2017). Our results suggest that individuals with high Aβ levels are more vulnerable to atrophy if the pathology is coupled with low baseline DMN connectivity.

Within the high Aβ group, baseline DMN connectivity remained significantly associated with DMN cortical thinning after controlling for functional coherence in the FPCN or longitudinal atrophy in the FPCN. The persistence of the DMN effect in the high amyloid group, despite covarying for other strongly associated cognitive networks, indicates a degree of specificity for DMN functional network connectivity to predict the rate of future atrophy within the default mode network. This provides partial support for the general hypothesis that neurons that fire together and wire together also die together, as illustrated by Seeley et al. (2009). These results are also congruent with previous work implicating the DMN’s particular vulnerability to AD-related pathology in early stages of Alzheimer’s disease relative to other cognitive networks such as the FPCN (Chhatwal et al., 2018). The DMN’s central role in cognition and memory may predispose it to a broad range of pathologies that affect brain networks in normal aging, but especially in disease (Jones et al., 2011). It should be noted, however, that our analyses within the high Aβ to understand DMN specificity were exploratory, and interpretations with these results should be approached with caution.

Previous reports implicate disrupted connectivity within the DMN as a reflection of accumulating biomarkers (Mormino et al., 2011, Jones et al., 2016). As previously stated, our results don’t show a baseline effect of Aβ on DMN connectivity, and our data do not align with the notion that amyloid pathology drives network failure or that atrophy is purely a product of tau pathology. These biomarkers may then contribute separately to longitudinal atrophy. Our analyses, however, do implicate that network disruption in the DMN precedes structural changes. More research is required to delve into the question if the high-DMN/high Aβ group reflects an excitotoxic or compensatory hyperconnectivity phase prior to DMN functional disruption.

In order to further understand the relationship between connectivity, Aβ, and selective atrophy, future studies could investigate other variables that could influence resilience and are related to functional connectivity, such as cognitive reserve, longitudinal tau-PET, cardiovascular risk, lifestyle, or genetic factors (Pietzuch et al., 2019). Including years of education did not significantly attenuate the observed effect in our models, however there could be other factors contributing to cognitive reserve in the high-DMN/high-Aβ group. Cardiovascular risk factors are also associated with rates of cognitive decline and cortical thinning (Rabin et al., 2019). Since we do not have tau-PET measures available at baseline, we cannot fully examine how tau-PET influences thinning from the time of the resting state measurements. It remains to be seen how these multiple facets are related to functional connectivity and atrophy in the presence of Aβ.

While there has been recent work (Ahmed et al., 2014, Calafate et al., 2015, Franzmeier et al., 2020, Hoenig et al., 2018, Vogel et al., 2020) and interest regarding the hypothesis that tau pathology propagates via networks (functionally or otherwise defined), our data and results are not suitable for assessing questions of the network spread of tau. We believe no observational study, even with longitudinal data, can discriminate network spread versus shared vulnerability to a given pathology or disease process. The general hypothesis that diseases can have network specific effects is not dependent on a particular pathology, process, or etiology. We know that brain networks exhibit similarities across many different biological scales (e.g. gene expression, metabolic processes, receptor distributions, evolutionary stage, etc…), which means diseases can show network specificity through any number of mechanisms or more generally via shared vulnerability across the different regions of a network. We also know that proper network functioning is a product of the constituent parts meaning pathology in one specific region will have repercussions for the functioning and health of the network as a whole. While we have demonstrated that there does appear to be a DMN specific effect amongst high amyloid individuals that is not accounted for by the FPCN or tau pathology, the mechanism of this effect remains unclear.

Better understanding the longitudinal trajectory of DMN fcMRI in the high-Aβ group is also intriguing, as recent longitudinal fcMRI work finds impaired individuals with high amyloid experience longitudinal degradation of the DMN and Salience networks (Schultz et al., 2019). A prior report also suggests that functional connectivity may paradoxically increase during certain early stages of disease (Schultz, et al., 2017), and the high-DMN/high-PiB group might indicate an earlier stage of disease relative to the low-DMN/high-PiB counterparts, and may relate to some set of compensatory mechanisms in the presence of amyloid pathology. With available diagnoses from neuropsychological follow-up of this sample, 14 of 120 participants progressed to MCI or dementia. 28.6% of those progressors were in the high-DMN/high-PiB group, while 50% of progressors were in the low-DMN/high-PiB group. The remaining 21.4% of progressors were in the low-PiB group. It is unclear if DMN functional connectivity is fully capturing a unique factor of decline related to AD progression, but this description further supports the findings of Buckley and colleagues (2017) implicating worse cognitive outcomes for low-DMN/high-PiB individuals.

Our sample from the Harvard Aging Brain Study is comprised of healthy, well-educated, elderly individuals who were cognitively normal at baseline. The DMN effect we report was only observed in those individuals with elevated amyloid burden at baseline, suggesting that the effect we report is specific to preclinical and early prodromal Alzheimer’s disease. In addition, while our overall sample size was large, the effect was carried by the smaller high-PiB group (N = 33). It will be important to replicate these results in other samples.

### 4.2 Conclusions

In summary, we have presented evidence that cortical thinning in the DMN is associated with a combination of greater Aβ burden and lower DMN functional connectivity at baseline in clinically-normal older adults. By using longitudinal structural MRI, we observed that neurodegeneration in the DMN in clinically-normal adults is associated with elevated Aβ pathology and moderated by cross-sectional DMN functional integrity, providing support for the hypothesis that functional network disruption or impairment presages atrophic processes within the regions and circuits that define the network. Although we did not observe this effect in the low-Aβ group, the lack of a finding may be due to the relative lack of pathologic heterogeneity and good health in our population. Our results encourage further investigation of longitudinal atrophy, particularly in regard to a temporal sequencing with functional connectivity, to better understand the progression of preclinical AD.

## CRediT authorship contribution statement

**Olivia L. Hampton:** Conceptualization, Data curation, Formal analysis, Methodology, Visualization, Writing - original draft, Writing - review & editing. **Rachel F. Buckley:** Conceptualization, Data curation, Formal analysis, Methodology, Visualization, Writing - original draft, Writing - review & editing. **Lyssa K. Manning:** Validation, Writing - review & editing. **Matthew R. Scott:** Validation, Writing - review & editing. **Michael J. Properzi:** Validation, Writing - review & editing. **Cleofé Peña-Gómez:** Validation, Writing - review & editing. **Heidi I.L. Jacobs:** Validation, Writing - review & editing. **Jasmeer P. Chhatwal:** Validation, Writing - review & editing. **Keith A. Johnson:** Funding acquisition, Writing - review & editing. **Reisa A. Sperling:** Funding acquisition, Writing - review & editing. **Aaron P. Schultz:** Conceptualization, Data curation, Formal analysis, Methodology, Visualization, Writing - original draft, Writing - review & editing.

## Declaration of Competing Interest

Dr. Johnson has served as a paid consultant for Bayer, GE Healthcare, Janssen, Siemens Medical Solutions, Genzyme, Novartis, Biogen, Roche, AZTherapy, GEHC, Lundbeck, Genentech, Lilly/Avid, AC Immune, and Abbvie. DDr. Sperling has received grants from National Institute on Aging, Alzheimer’s Association, Eli Lilly and Co., Janssen Pharmaceuticals, and Eisai. She has consulted for AC Immune, Eisai, Janssen, Prothena, Roche, and Takeda. There are no other conflicts of interest to be reported.
